# Sodium–glucose cotransporter-2 inhibitors in heart failure patients across the range of body mass index: a systematic review and meta-analysis of randomized controlled trials

**DOI:** 10.1007/s11739-024-03532-8

**Published:** 2024-02-14

**Authors:** Anastasia Adamou, David Dimitris Chlorogiannis, Ioannis G. Kyriakoulis, Iliana Stamatiou, Despoina Koukousaki, Ioannis Kardoutsos, Dimitrios Sagris, Wolfram Doehner, George Ntaios

**Affiliations:** 1 Department of Internal Medicine, Faculty of Medicine, School of Health Sciences, University of Thessaly, 41110 Larissa, Thessaly Greece; 2 Department of Radiology, Brigham and Women’s Hospital, Boston, MA USA; 3 Department of Internal Medicine, University Hospital of Alexandroupolis, Alexandroupolis, Greece; 4 Berlin Institute of Health Center for Regenerative Therapies (BCRT), Berlin, Germany; 5 Department of Cardiology (Virchow Klinikum), German Centre for Cardiovascular Research (DZHK), Partner Site Berlin, Universita¨Tsmedizin, Berlin, Germany; 6 Center for Stroke Research Berlin, Charite Universitatsmedizin Berlin, Berlin, Germany

**Keywords:** SGLT2, BMI, HF, Ejection fraction

## Abstract

Sodium–glucose cotransporter-2 (SGLT2) inhibitors improve outcomes in patients with heart failure, with or without diabetes. We sought to assess whether there is an interaction of these effects with body mass index (BMI). A systematic review of the MEDLINE and Scopus databases (last search: November 15th, 2022) was performed according to the PRISMA statement. Studies eligible for this review were randomized control trials (RCTs) with patients with chronic heart failure with either preserved or reduced ejection fraction randomly assigned to SGLT2 inhibitors or placebo. Data were extracted independently by two reviewers. BMI was classified according to the WHO classification into under/normal weight (BMI: < 25 kg/m^2^), overweight (BMI: 25–29.9 kg/m^2^), obesity class I (BMI: 30–34.9 kg/m^2^), and obesity classes II/III (BMI: ≥ 35 kg/m^2^). All analyses were performed using RevMan 5.4. Among 1461 studies identified in the literature search, 3 were eligible and included in the meta-analysis. Among 14,737 patients (32.2% were women), 7,367 were randomized to an SGLT2 inhibitor (dapagliflozin or empagliflozin) and 7,370 to placebo. There were significantly fewer hospitalizations for HF (OR: 0.70, 95%CI: 0.64–0.76), cardiovascular deaths (OR:0.86, 95%CI: 0.77–0.97) and all-cause deaths (OR:0.90, 95%CI: 0.82–0.98) in the SGLT2 inhibitors group compared to the placebo group, without any interaction with BMI group (test for subgroup differences: *x*^2^ = 1.79, *p* = 0.62; *x*^2^ = 0.27, *p* = 0.97; *x*^2^ = 0.39, *p* = 0.94, respectively). There is no interaction between the efficacy of SGLT2 inhibitors and BMI in patients with HF with either preserved or reduced ejection fraction. SGLT2 inhibitors are associated with improved outcomes regardless of the BMI.

Trial registration: PROSPERO ID: CRD42022383643.

**Supplementary Information:**

The online version contains supplementary material available at 10.1007/s11739-024-03532-8.

## Introduction

Sodium–glucose cotransporter 2 inhibitors (SGLT2i) constitute one of the main pillars of treatment in patients with heart failure, as they improve survival and delay the progression of the disease [1]. SGLT2i balance glucose levels by blocking tubular reabsorption, enhance natriuresis, cause intravascular volume contraction, and alter intra-renal hemodynamics, which likely contribute to beneficial effects on regulating blood pressure, mild weight loss, and albuminuria [2]. In addition, SGLT2i are associated with mild weight loss [3].

The association between body mass index (BMI) and HF is heterogenous. Obesity has a high prevalence in patients with heart failure, and almost half of the patients with heart failure with preserved ejection fraction have a BMI ≥ 30 kg/m^2^ [4, 5]. There is evidence of slower progression of the disease and better clinical outcomes in patients with higher BMI. The relation between BMI and outcomes in patients with heart failure is represented as a J-shape describing that the patients in the BMI range of 25–35 kg/m^2^ appear to have the lowest risk of mortality [5]. On the contrary, HF patients with normal or low BMI or with weight loss during their disease experience more hospitalizations and higher mortality [6, 7]. This phenomenon is described as “obesity–survival paradox”.

In this context, concern was raised whether patients with heart failure and increased BMI would still benefit from SGLT2i [8]. To address this question, we performed a systematic review and meta-analysis of randomized controlled trials of SGLT2 inhibitors in patients with heart failure to assess whether their beneficial effect is consistent across the range of BMI.

## Methods

This systematic review was performed according to the updated Preferred Reporting Items for Systematic reviews and Meta-Analyses statement (PRISMA) [13] and was registered at PROSPERO (CRD42022383643). Institutional board review approval is not required for a study-level systematic review.

### Literature search

We searched PubMed and Cochrane Library literature from inception until November 15, 2022, confined to studies in English language using the following keywords: (SGLT-2 OR "sodium–glucose transport protein 2" OR dapagliflozin OR empagliflozin OR canagliflozin OR ertugliflozin) AND (heart OR cardiac) AND (BMI OR obes* OR weight OR "body mass") AND (randomized OR trial). The systematic search was conducted by two independent investigators, blind to each other, and any discrepancies were resolved by consensus between them.

### Eligibility criteria

Studies were eligible for inclusion if they were randomized controlled trials (RCTs) of SGLT2 inhibitors compared to placebo in patients with heart failure with preserved or reduced injection fraction and provided results stratified by body mass index (BMI). We classified BMI according to the WHO classification: normal weight (< 25 kg/m^2^), overweight (BMI: 25–29.9 kg/m^2^), obesity class I (BMI: 30–34.9 kg/m^2^), obesity class II/III (BMI: ≥ 35 kg/m^2^). Review articles, case series, and case report were excluded. The primary outcome was cardiovascular mortality. The secondary outcomes were hospitalization for heart failure and all-cause mortality.

### Selection of eligible studies

Titles, abstracts, and keywords of all the articles were screened by two independent reviewers and irrelevant reports were removed. Full text screening of the selected articles was performed by the two same reviewers. Disagreements were resolved by consensus.

### Data extraction

A data extraction form was created to extract the study’s pre-determined characteristics and outcomes of interest. Study characteristics included authorship, name of the clinical trial, year of publication, study size, participants per interventional arm, and study duration including follow-up. Population characteristics included mean age, sex, race, mean BMI at baseline, comorbidities, patients' standard of care, and mean left ventricular ejection fraction. Suitability of the form was evaluated in two randomly selected studies by all study’s authors. After form finalization, two of the authors extracted the data from each study.

### Risk of bias assessment

The revised Cochrane ‘Risk of bias tool for randomized trials’ (RoB 2.0) was used to assess the risk of bias of the included studies [9]. Three independent investigators (IK, DK, and IS) applied the tool to each study and examined the five domains that RoB 2.0 addresses: (i) bias arising from the randomization process, (ii) bias due to deviations from intended interventions, (iii) bias due to missing outcome data, (iv) bias in measurement of the outcome, and (v) bias in selection of the reported result. Any discrepancy or uncertainty was resolved by consensus discussion among all authors. The same three investigators independently assessed the certainty of evidence and evaluated the quality of the body of evidence using the GRADE approach (Grading of Recommendations Assessment, Development and Evaluation) [10]. Criteria that downgrade the certainty of evidence (risk of bias, publication bias, inconsistency, indirectness, and imprecision of results) and factors that upgrade it (large effect size, dose response, and the effect of plausible residual confounding) were used for characterization of the certainty of evidence as high, moderate, low, or very low. Publication bias was assessed graphically using funnel plots.

### Statistical analysis

Continuous variables were summarized by mean and standard deviation (SD), and categorical variables by relative frequency and percentage. The Wan et al. method was used to estimate the means and SDs of continuous variables whenever medians and ranges and median and interquartile ranges were provided, respectively [11]. Odds ratio with 95% confidence intervals (CIs) were used as measure of effect. A random-effect model (Mantel–Haenzel procedure) was used to estimate the pooled OR [12]. Inverse-variance weights were used in all cases. Inconsistency test (*I*^2^) statistics were used to assess the heterogeneity (*I*^2^ = 100% × (*Q* − df)/Q, where *Q* = χ^2^ (Cochran’s heterogeneity statistic) and df = degrees of freedom), where *I*^2^ ≤ 25% signifies low heterogeneity, I^2^ ≤ 50% is moderate heterogeneity and *I*^2^ > 50% is considered high heterogeneity [13]. *p* values < 0.05 were considered significant. Leave-one-out analysis was performed by removing one study at a time and repeating the statistical analysis. Review Manager software version 5.4.1 (Cochrane Collaboration) "metafor" package using R Studio Version 2023.09.1 + 494 (2023.09.1 + 494) was used for the analyses [14]. To determine if our four BMI groups were significantly different from each other on the mean LVEF (left ventricular ejection fraction) and no cross-interaction between them was present, one-way ANOVA test was performed.

## Results

### Study and patient characteristics

**Fig. 1:**
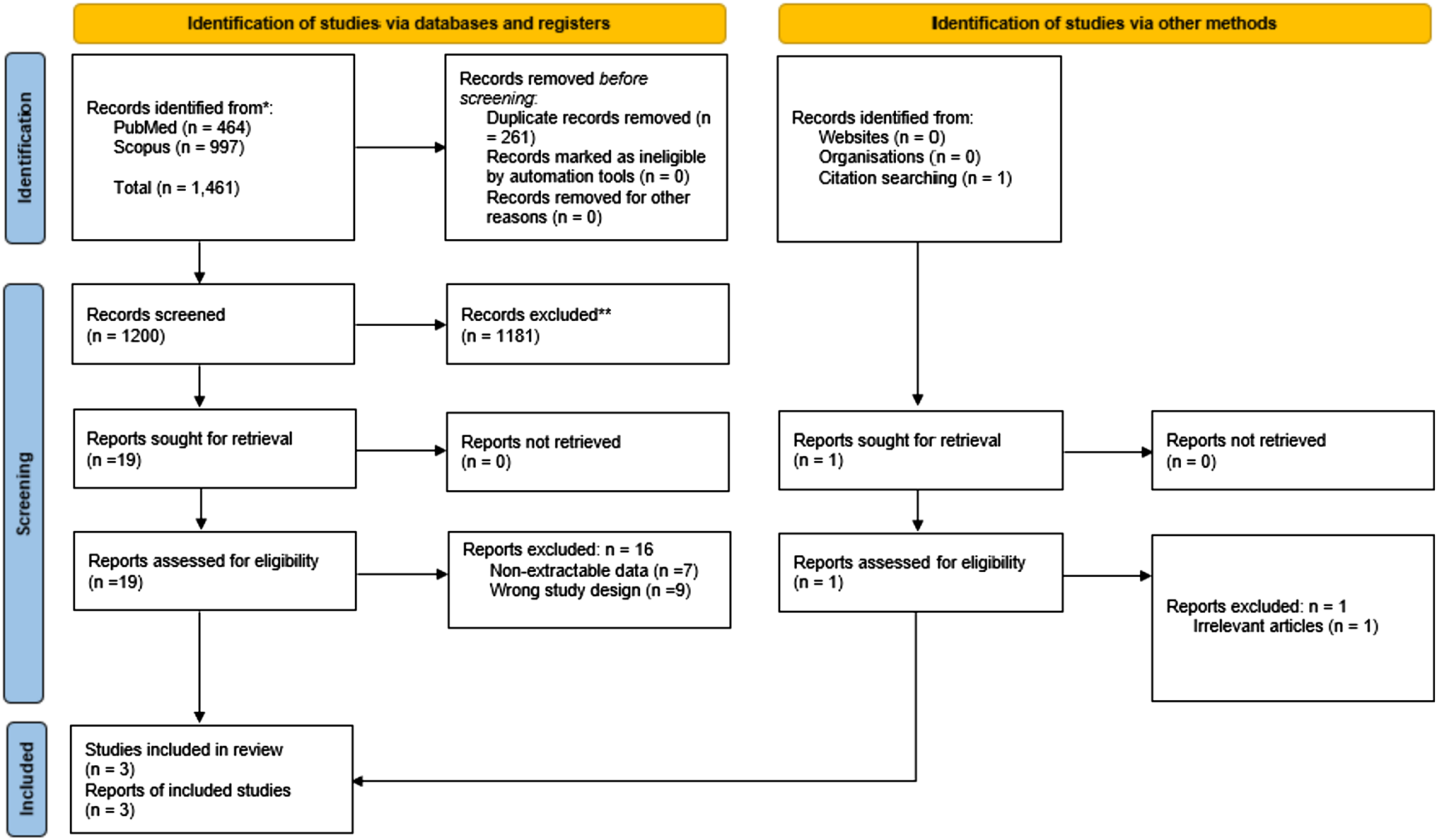
PRISMA flowchart

The systematic search identified 1461 articles for potential inclusion. After title and abstract screening, 19 were deemed eligible for full text screening, and three were finally included in the meta-analysis [8, 20, 21] (Fig. 1).

The three studies included 14,737 patients with 7367 (49.9%) patients randomized to a SGLT2 inhibitor (dapagliflozin or empagliflozin) and 7370 (50.1%) to placebo. In the two trials, the DELIVER [15] (*n* = 6263) and DAPA-HF [16] (*n* = 4744), participants were randomly assigned to dapagliflozin or placebo and in the other trial, EMPEROR-reduced [8] (*n* = 3730), participants were randomly assigned to empagliflozin or placebo. DAPA-HF and EMPEROR-reduced included patients with heart failure with reduced ejection fraction as DELIVER participants were patients with heart failure with preserved ejection fraction. The mean age was 68.4 ± 6.9 years with male predominance (67.8%). Patient characteristics are summarized in Table 1.

**Table 1:** Patient characteristics

BMI		< 25 kg/m^2^			25–29.9 kg/m^2^			30–34.9 kg/m^2^			≥ 35 kg/m^2^		
study		DELIVER	DAPA-HF	EMPEROR-R	DELIVER	DAPA-HF	EMPEROR-R	DELIVER	DAPA-HF	EMPEROR-R	DELIVER	DAPA-HF	EMPEROR-R
n	*SGLT2i*	709(50.8%)	672(49.9%)	599(49.2%)	1025(49.4%)	865(50.2%)	664(49.4%)	772(49%)	512(50.5%)	406(52.5%)	623(51.4%)	322(48.9%)	194(49.4%)
	*Placebo*	688(49.2%)	676(50.1%)	619(50.8%)	1048(50.6%)	857(49.8%)	681(50.6%)	802(51%)	501(49.5%)	368(47.5%)	590(48.6%)	337(51.1%)	199(50.6%
Age		73.4 ± 10	67 ± 12	67.8 ± 11.4	72.4 ± 9.5	67 ± 10	67.6 ± 10.6	71.4 ± 9	66 ± 10	65.4 ± 10.5	68.8 ± 9.1	63 ± 11	64 ± 11.7
Female		603(43.2%)	334(24.8%)	302(24.8%)	828(39.9%)	346(20.1%)	281(20.9%)	676(42.9%)	227(22.4%)	182(23.5%)	635(52.3%)	202(30.7%)	128(32.6%)
Asian		669(49.7%)	656(48.7%)	442(36.3%)	453(21.9%)	349(20.3%)	173(12.9%)	119(7.6%)	87(8.6%)	45(5.8%)	32(2.6%)	24(6.7%)	12(3.1%)
Black		25(1.8%)	47(3.5%)	80(6.6%)	47(2.7%)	69(4%)	96(7.1%)	32(2%)	58(5.7%)	48(6.2%)	55(4.5%)	52(7.9%)	33(8.3%)
White		601(43%)	621(46.1%)	638(52.4%)	1427(68.8%)	1283(74.5%)	1018(75.7%)	1333(84.7%)	854(84.3%)	643(83.1%)	1073(88.5%)	573(86.9%)	330(84%)
NYHA III/IV		287(20.5%)	374(27.7%)	271(22.3%)	439(21.2%)	528(30.7%)	297(22.1%)	414(26.3%)	362(35.7%)	221(28.6%)	408(33.6%)	263(39.9%)	141(35.9%)
Diabetes		434(31.1%)	431(32%)	503(41.3%)	885(42.7%)	665(38.6%)	661(49.1%)	782(49.7%)	500(49.4%)	452(58.4%)	703(58%)	387(58.7%)	240(61.1%)
Hypertension		1113(79.7%)	835(61.9%)	NA	1812(87.4%)	1277(74.2%)	NA	1474(93.6%)	836(82.5%)	NA	1148(94.6%)	572(86.8%)	NA
Atrial fibrillation		751(53.8%)	435(32.3%)	403(33.1%)	1060	672(39%)	488(36.3%)	929(59%)	406(40.1%)	308(39.8%)	486(40.1%)	305(46.3%)	170(43.3%)
LVEF		53.2 ± 10.1	30 ± 7	27.4 ± 6.2	53 ± 10	31 ± 7	27.5 ± 6	54 ± 10	32 ± 7	27.6 ± 5.8	54.3 ± 9.4	31 ± 7	27.3 ± 6.2
Prior HHF		611(43.7%)	656(48.7%)	387(31.8%)	817(39.4%)	827(48%)	394(29.3%)	625(39.7%)	462(45.6%)	234(30.2%)	482(39.7%)	305(46.3%)	136(34.6%)
Beta-blockers		1074(76.9%)	1254(93%)	1128(92.6%)	1694(81.7%)	1663(96.6%)	1283(95.4%)	1353(86%)	987(97.4%)	744(96.1%)	1051(86.6%)	652(98.9%)	378(96.2%)
ACEs or ARBs		971(70%)	1227(91%)	809(66.4%)	1594(76.9%)	1638(95.1%)	1225(91.1%)	1289(81.9%)	973(96.1%)	719(92.9%)	973(80.2%)	636(96.5%)	361(91.9%)
MRAs		642(46%)	961(71.3%)	851(69.9%)	869(41.9%)	1209(70.2%)	944(70.2%)	679(43.1%)	735(72.6%)	561(72.5%)	475(39.2%)	465(70.6%)	305(77.6%)
ICD		NA	261(19.4%)	285(23.4%)	NA	467(27.1%)	453(33.7%)	NA	302(29.8%)	288(37.2%)	NA	210(31.9%)	144(36.6%)

Data are presented as mean ± SD for continuous measures, and *n* (%) for categorical measures. Abbreviations: BMI: body mass index, SGLT2i: sodium–glucose cotransporter 2 inhibitors, NYHA III/IV: New York Heart Association classification III/IV, LVEF: left ventricular ejection fraction, HHF: hospitalization due to heart failure, ACEs: angiotensin-converting enzyme inhibitors, ARBs: angiotensin receptor blockers, MRAs: mineralocorticoid receptor antagonists, ICD: implantable cardioverter–defibrillator

### Assessment of quality of included studies

All studies were evaluated as having low risk of bias. The outcomes of Rob 2.0 evaluation are presented in Appendix. No outcome demonstrated statistically significant heterogeneity (Appendix, Figure S1). The funnel plot’s symmetric distribution of the mean effect size for all outcomes indicates low risk of publication bias of the included studies (Appendix, Tables S1–S3).

### Cardiovascular mortality

**Fig. 2:**
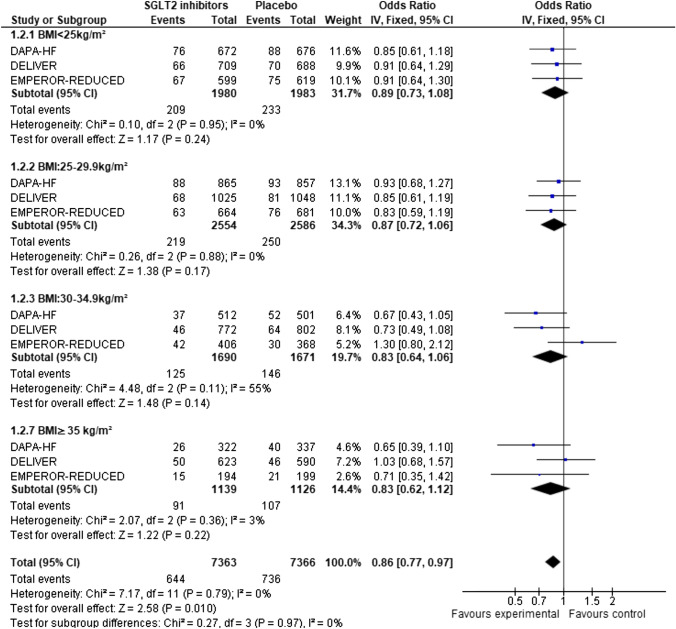
Forest plot of odds ratios (ORs) with pooled effect size and associated 95% confidence interval (CI) reported for cardiovascular mortality stratified by BMI

Among the 14,737 patients who were recruited in the three included studies, randomization to SGLT2 inhibitor was associated with a 14% reduction in cardiovascular mortality (OR: 0.86, 95%CI: 0.77–0.97) compared to placebo, without any interaction with BMI (test for subgroup differences: *x*^2^ = 0.16, *p* = 0.98 and after inverse-variance weighted regression analysis *p* = 0.63). The absolute risk reduction was − 0.01 (OR: − 0.02, − 0.00) (Appendix, Figure S5). The numbers needed to treat (NNT) to prevent one cardiovascular death was 83.3 in the normal weight group, 90.9 in the overweight group, 74.6 in the obesity I group, and 66.7 in the obesity II group. There was no statistically significant heterogeneity between studies (*p* = 0.79) (Fig. 2).

### All-cause mortality

**Fig. 3:**
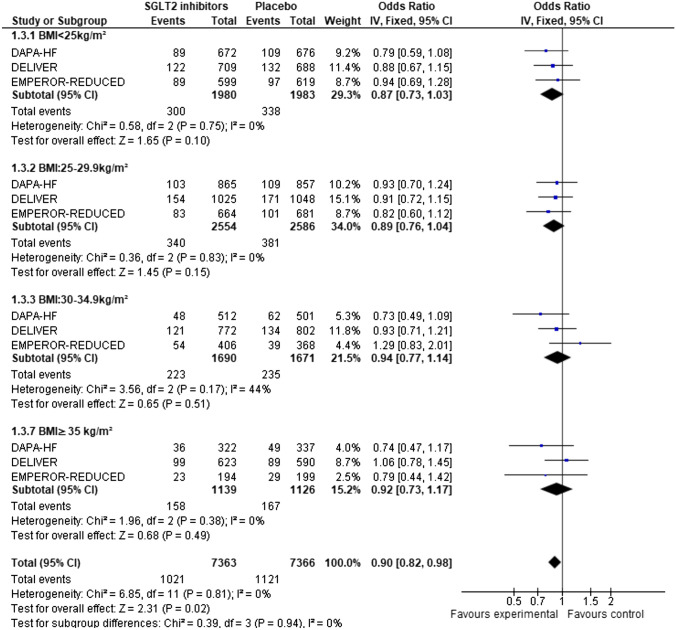
Forest plots of odds ratios (ORs) with pooled effect size and associated 95% confidence interval (CI) reported for all-cause mortality stratified by BMI

Among the 14,737 patients who were recruited in the three included studies, randomization to SGLT2 inhibitor was associated with a 10% odds reduction in all-cause mortality (OR: 0.90, 95%CI: 0.82–0.98) compared to placebo, without any interaction with BMI (test for subgroup differences: x^2^ = 0.34, *p* = 0.95 and after inverse-variance weighted regression analysis *p* = 0.57). The absolute risk reduction was − 0.01 (OR: − 0.02, -− .00) (Appendix, Figure S6). The NNT to prevent one all-cause death was 52.9 in the normal weight group, 70.4 in the overweight group, 114.9 in the obesity I group, and 104.2 in the obesity II group. There was no statistically significant heterogeneity between studies (*p* = 0.81) (Fig. 3).

### Hospitalization for heart failure

**Fig. 4:**
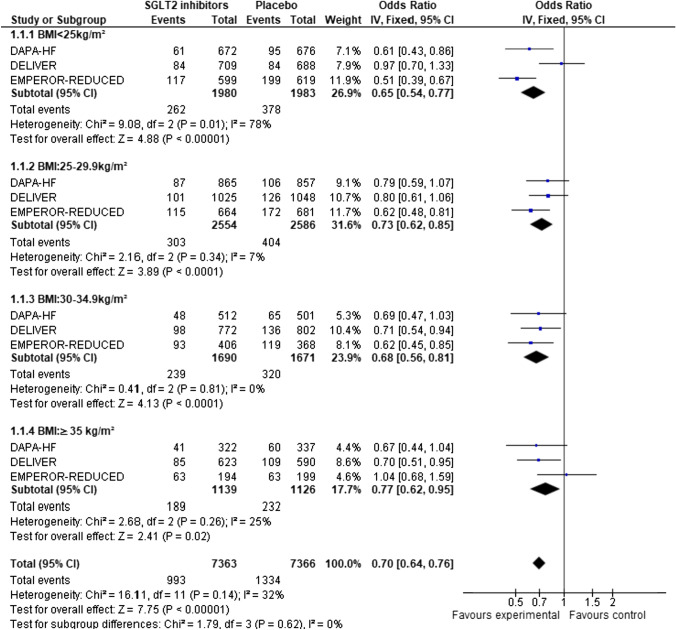
Forest plots of odds ratios (ORs) with pooled effect size and associated 95% confidence interval (CI) reported for hospitalization events for HF, stratified by BMI

Among the 14,737 patients who were recruited in the three included studies, randomization to SGLT2 inhibitor was associated with a 30% odds reduction in hospitalization for HF (OR: 0.70, 95%CI: 0.64–0.76), without any interaction with BMI (test for subgroup differences: *x*^2^ = 0.86, *p* = 0.83, and after inverse-variance weighted regression analysis *p* = 0.23). The absolute risk reduction was − 0.04 (OR: − 0.05, − 0.03) (Appendix, Figure S7). The NNT to prevent one HF hospitalization was 16.9 in the normal weight group, 27 in the overweight group, 20 in the obesity I group, and 24.9 in the obesity II group. There was no statistically significant heterogeneity between studies (*p* = 0.14) (Fig. 4).

### Cross-interaction between ejection fraction and BMI

Across the four different BMI groups included in this meta-analysis, there was no variance concerning the ejection fraction for each group. The results from the one-way ANOVA test revealed a *p* value = 0.99) (Appendix, Table S1).

### Patients with heart failure with reduced ejection fraction.

Further analysis was performed separately in the two trials with patients with heart failure with reduced ejection fraction. Among the 8474 patients of the DAPA-HF and the EMPEROR-reduced trials, no interaction with BMI was observed in cardiovascular mortality (test for subgroup differences: *x*^2^ = 1.53, *p* = 0.67) (Appendix, Figure S8), all-cause mortality (test for subgroup differences: *x*^2^ = 0.89, *p* = 0.83) (Appendix, Figure S9), and hospitalization events for heart failure (test for subgroup differences: *x*^2^ = 5.73, *p* = 0.13) (Appendix, Figure S10) [8, 16].

## Discussion

This systematic review and meta-analysis of 14,737 HF patients with preserved or reduced EF shows that the beneficial effect of empagliflozin and dapagliflozin on cardiovascular mortality, all-cause mortality, and HF hospitalization is consistent across the BMI range.

Τhis observation is consistent with the results of clinical trials of SGLT2 inhibitors in other populations such as those with chronic renal disease or type II diabetes mellitus, in which there was no interaction between the effect and BMI [17, 18]. The totality of this evidence in all three therapeutic areas of SGLT2 inhibitors indicates that there is no reason to consider BMI as a treatment-modifying factor in the decision to start dapagliflozin or empagliflozin in an eligible patient, regardless of the underlying indication.

SGLT2 inhibitors are associated with a minor loss of body weight; however, this class should not be considered as a tool for primary weight loss management in patients with high or very high BMI, as the associated weight reduction was less than 5%. This weight reduction was shown to be higher with increasing BMI [5]. In patients with HF and reduced ejection fraction, the weight loss in empagliflozin-treated patients, especially when unintentional, was associated with higher risk of all-cause mortality, which is consistent with the obesity paradox that has been described in heart failure as well as in other cardiovascular diseases [19–21]. However, the presence of this association also in placebo-treated patients and the persistent beneficial effect of SGLT2 inhibitors across the BMI range indicate that the weight loss in empagliflozin- and dapagliflozin-treated patients is not related to the observed obesity paradox [8]. The association of cardiovascular outcomes with obesity in patients with cardiovascular disease needs further research. Recent evidence indicates that the use of other anthropometric indices, such as the waist-to-height ratio, which do not incorporate weight and better reflect the location and amount of ectopic fat, might be more informative [22, 23].

## Limitations

A limitation of the present analysis is the inclusion of only three studies. However, these were prospective randomized controlled trials and included > 14,000 patients. Another limitation is that the quantitative analysis was performed on study-level data rather than in patient-level data which were not available. An individual patient data analysis could support an analysis using BMI as a continuous covariate and possibly provide a more accurate assessment of the effect. This restriction cannot also allow us to perform analysis in specific populations that are at high cardiovascular risk, such as patients with hypertension or diabetes. Also, some of the reported BMI subgroups were pooled into a single subgroup to facilitate a homogeneous reporting of the results. Finally, we could not study alternate indices of obesity like waist circumference, skinfold thickness, or dual energy X-ray absorptiometry, which could provide further insight given that they can assess body fat distribution more accurately [22, 23].

## Conclusion

In conclusion, this meta-analysis of randomized trials of SGLT2 inhibitors in patients with heart failure shows no interaction between the beneficial effect of dapagliflozin and empagliflozin on cardiovascular mortality, all-cause mortality and HF hospitalization, and BMI-defined classes of obesity.

### Supplementary Information

Below is the link to the electronic supplementary material.

Supplementary file1 (DOCX 315 KB)

## Data Availability

The datasets generated during and/or analysed during the current study are available from the corresponding author on reasonable request.
