# Supplementary material for: Sodium–glucose cotransporter-2 inhibitors in heart failure patients across the range of body mass index: a systematic review and meta-analysis of randomized controlled trials
Source: Intern Emerg Med. 2024 Feb 14;19(2):565–73. doi: 10.1007/s11739-024-03532-8 (PMC10955025; doi:10.1007/s11739-024-03532-8)
Supplement: Supplementary file 1 — Supplementary file1 (DOCX 315 KB) [file 11739_2024_3532_MOESM1_ESM.docx]

**Figure S1.** Risk of bias assessment


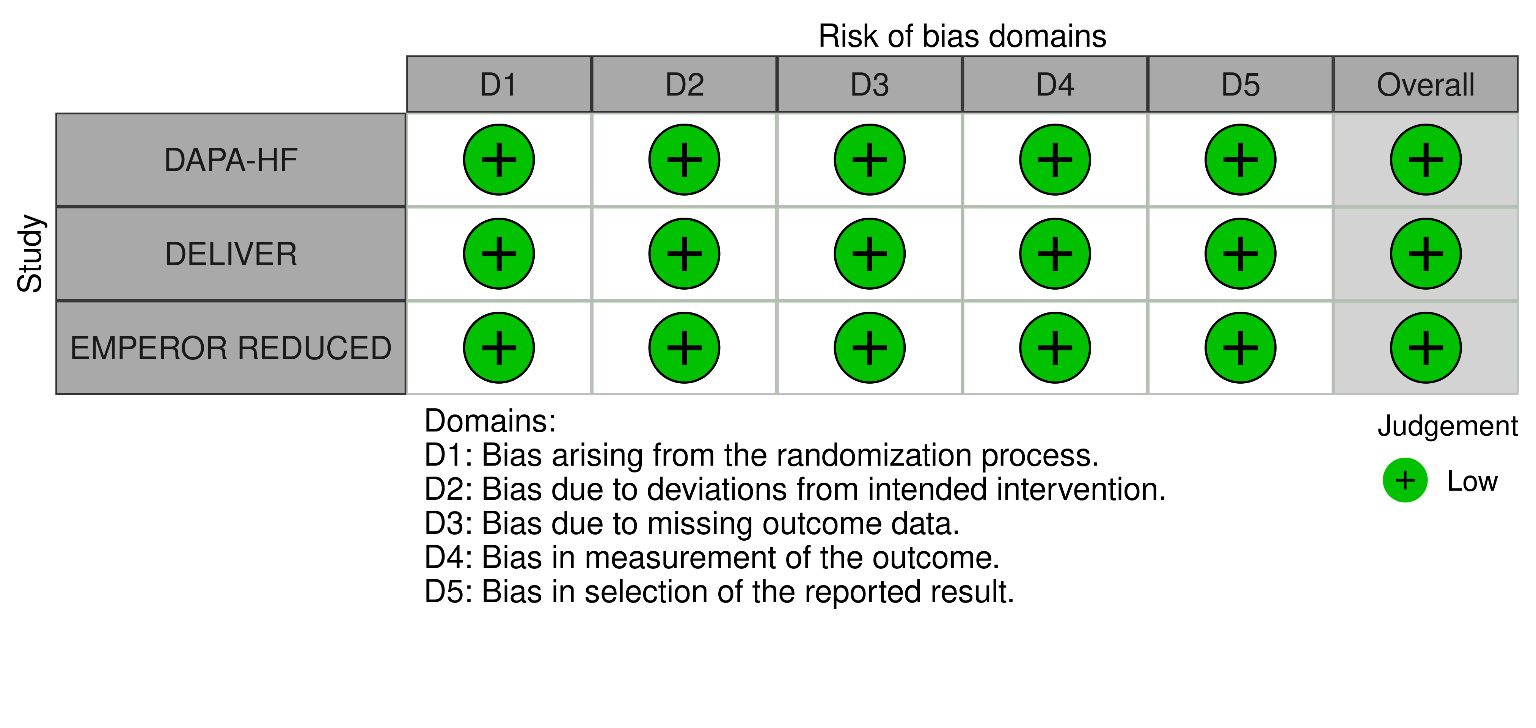


**Figure S2**: Funnel plot of symmetric distribution of the mean effect size for cardiovascular mortality stratified by BMI.


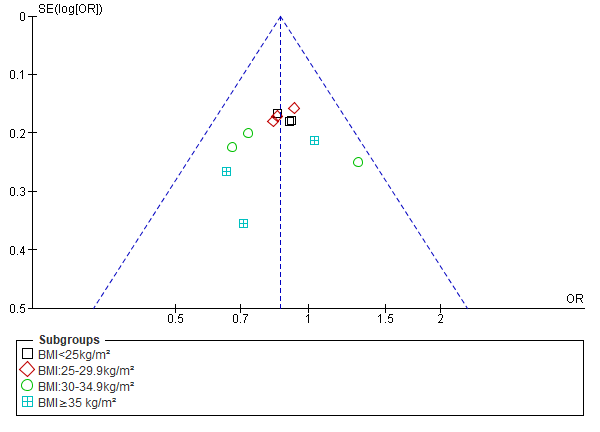


**Figure S3**: Funnel plot of symmetric distribution of the mean effect size for all-cause mortality stratified by BMI.


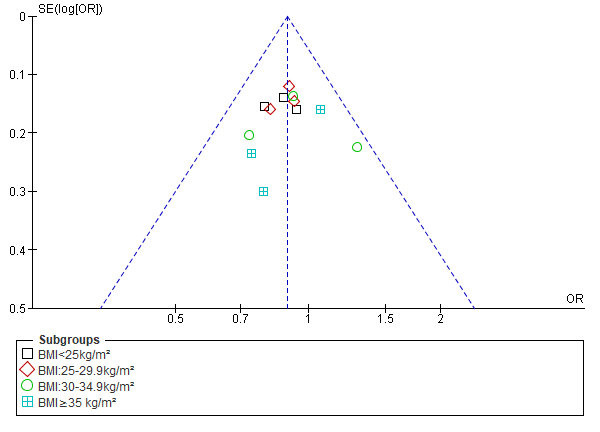


**Figure S4**: Funnel plot of symmetric distribution of the mean effect size for hospitalization events stratified by BMI.


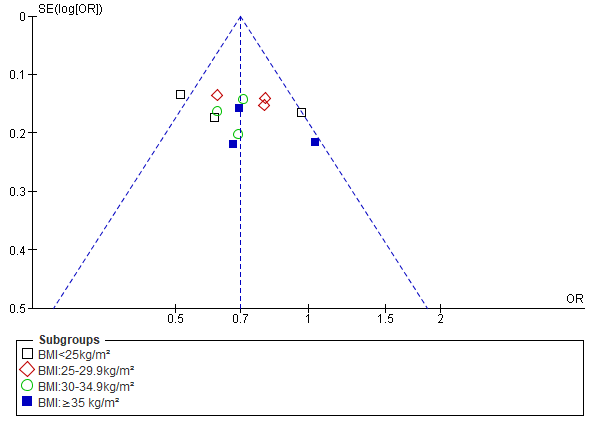


**Fig. S5.** Forest plots of absolute risk reduction (ARR) with pooled effect size and associated 95% confidence interval (CI) reported cardiovascular mortality, stratified by BMI


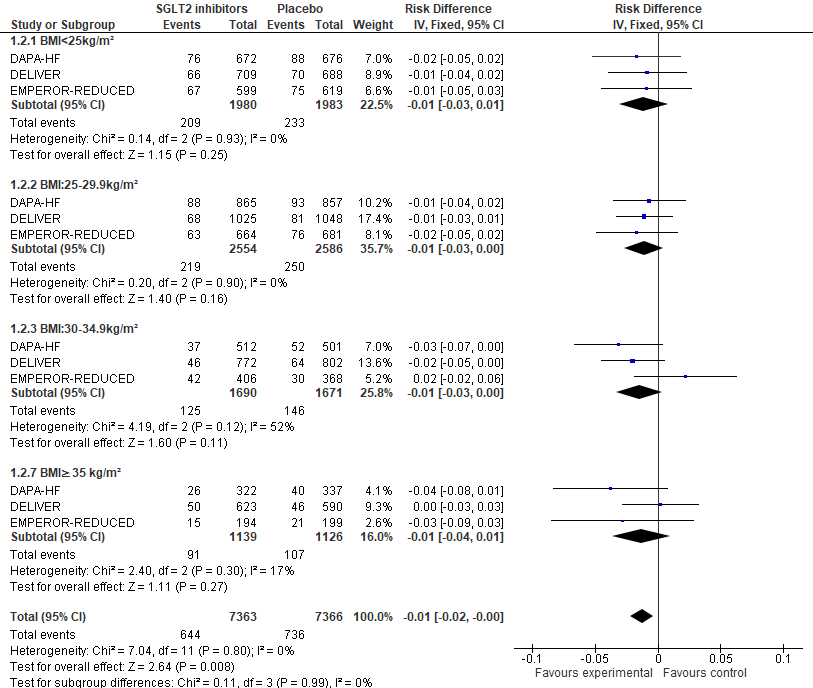


**Fig. S6.** Forest plots of absolute risk reduction (ARR) with pooled effect size and associated 95% confidence interval (CI) reported all-cause mortality, stratified by BMI


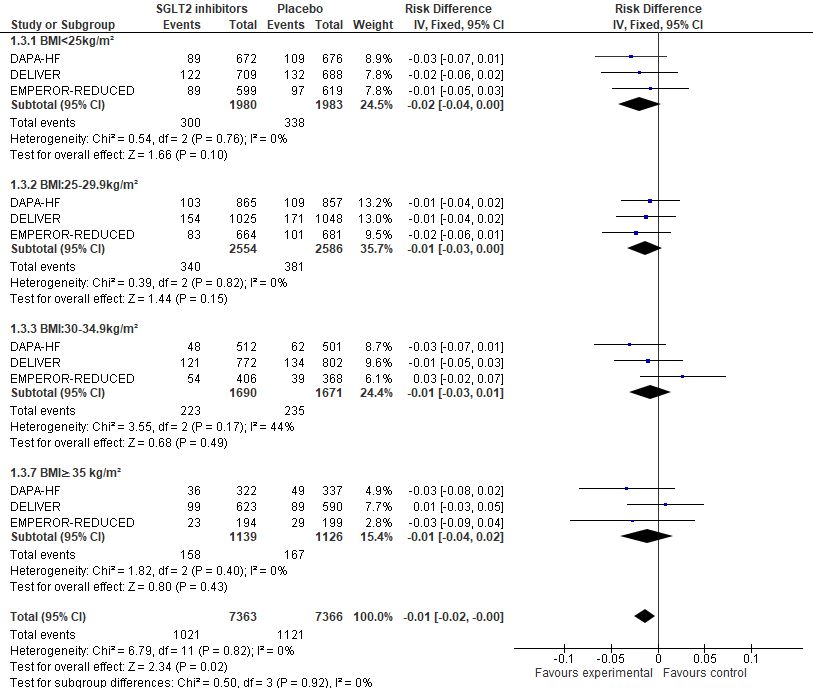


**Fig. S7.** Forest plots of absolute risk reduction (ARR) with pooled effect size and associated 95% confidence interval (CI) reported for hospitalization events for HF, stratified by BMI


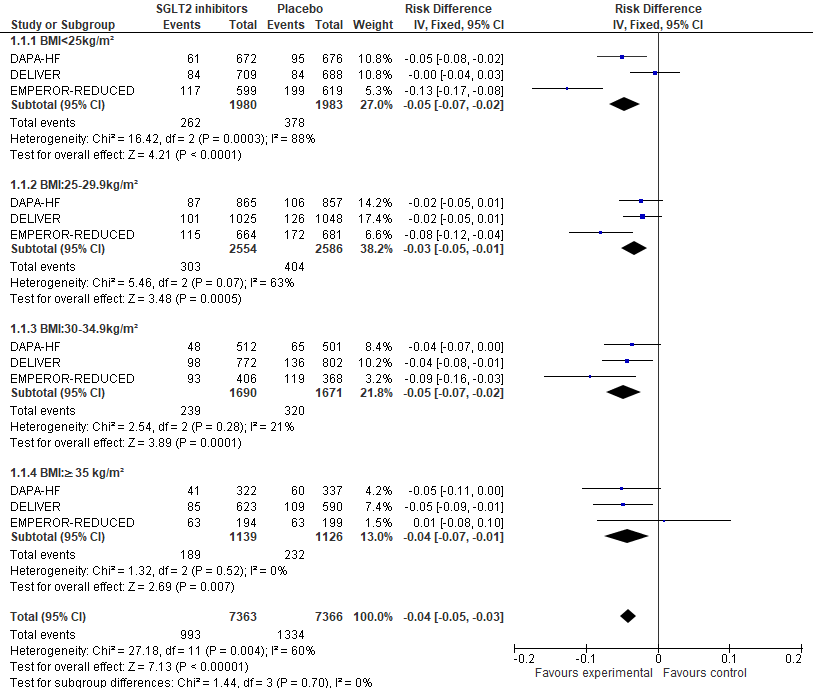


**Figure S8:** Forest plots of odds ratios (ORs) with pooled effect size and associated 95% confidence interval (CI) reported for cardiovascular mortality stratified by BMI for patients with heart failure with reduced ejection fraction.


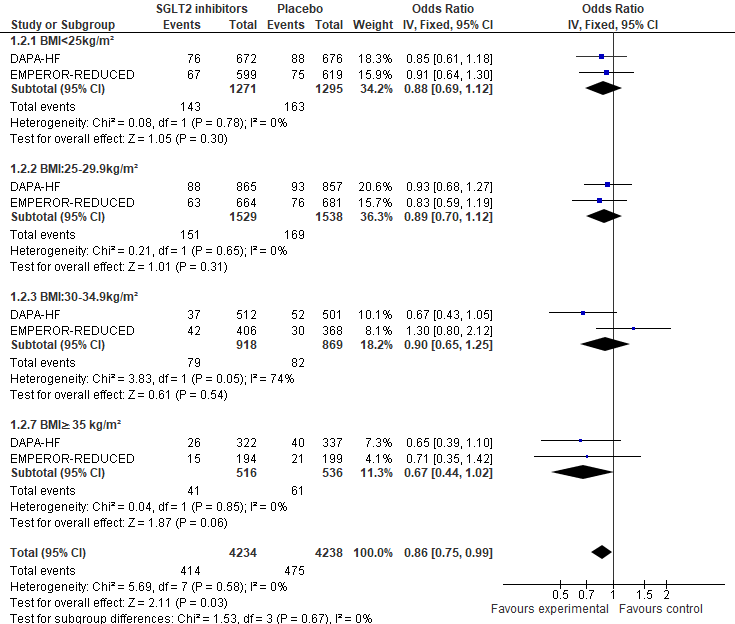


**Figure S9:** Forest plots of odds ratios (ORs) with pooled effect size and associated 95% confidence interval (CI) reported for all-cause mortality stratified by BMI for patients with heart failure with reduced ejection fraction.


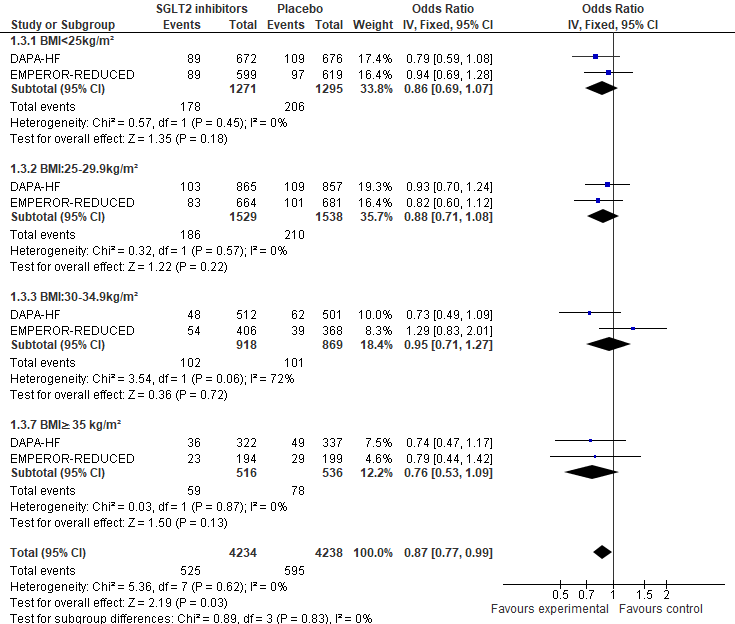


**Figure S10:** Forest plots of odds ratios (ORs) with pooled effect size and associated 95% confidence interval (CI) reported for hospitalization events for HF stratified by BMI only for patients with heart failure with reduced ejection fraction.


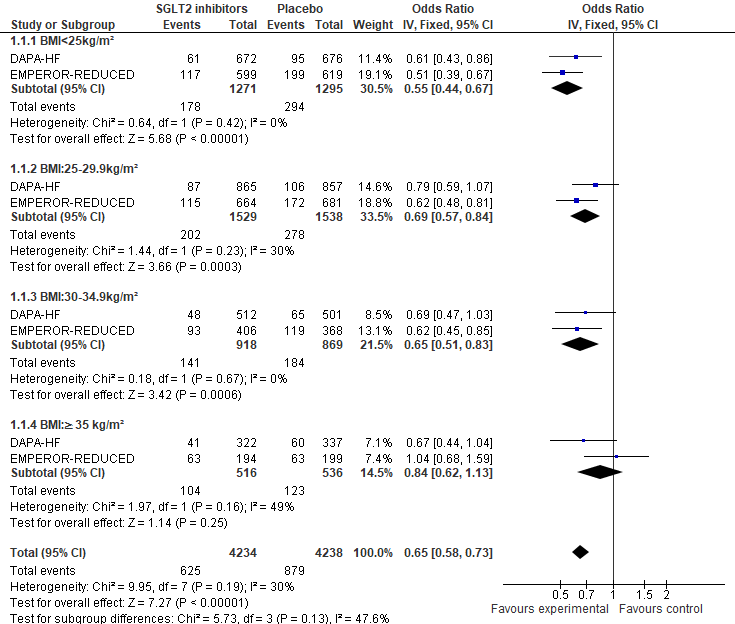


| SUMMARY |  |  |  |  |  |  |
| --- | --- | --- | --- | --- | --- | --- |
| *Groups* | *Count* | *Sum* | *Average* | *Variance* |  |  |
| meanLVEF_Under/Normalweight | 3 | 110,6 | 36,86667 | 201,7733 |  |  |
| meanLVEF_Overweight | 3 | 111,5 | 37,16667 | 191,0833 |  |  |
| meanLVEF_Obesity_Class_I | 3 | 113,6 | 37,86667 | 200,0533 |  |  |
| meanLVEF_Obesity_Class_II/III | 3 | 112,6 | 37,53333 | 214,2633 |  |  |
|  |  |  |  |  |  |  |
| ANOVA |  |  |  |  |  |  |
| *Source of Variation* | *SS* | *df* | *MS* | *F* | *P-value* | *F crit* |
| Between Groups | 1,7025 | 3 | 0,5675 | 0,002812 | 0,999776 | 4,066181 |
| Within Groups | 1614,347 | 8 | 201,7933 |  |  |  |
|  |  |  |  |  |  |  |
| Total | 1616,049 | 11 |  |  |  |  |

**Table S1:** ANOVA test results for cross-interaction evaluation between ejection fraction and the different BMI groups.
